# Clinical Image Quality and Reader Variability in 3D Synthetic Brain MRI Compared with Conventional MRI

**DOI:** 10.3390/tomography12020013

**Published:** 2026-01-23

**Authors:** Alexander von Hessling, Chloé Sieber, Maria Blatow, Christian Berner, Dirk Lehnick, Frauke Kellner-Weldon

**Affiliations:** 1 Section of Neuroradiology, Department of Radiology and Nuclear Medicine, Neurocenter, Cantonal Hospital Lucerne, University Teaching and Research Hospital, University of Lucerne, 6000 Lucerne, Switzerlandchristian.berner@spital-emmental.ch (C.B.); 2 Faculty of Health Sciences and Medicine, University of Lucerne, 6000 Lucerne, Switzerland; 3 Center for Clinical Research—Biostatistics and Methodology, Lucerne Cantonal Hospital, University of Lucerne, 6000 Lucerne, Switzerland

**Keywords:** synthetic magnetic resonance imaging, synthetic imaging, 3D synthetic imaging, 3D-QALAS, relaxometry, quantitative magnetic resonance imaging

## Abstract

**Background/Objectives**: This study evaluated the clinical image quality of three-dimensional synthetic MRI (3D SI) compared with conventional MRI (cMRI), focusing on tissue contrast, anatomical detail, and motion sensitivity. **Methods**: Patients with nonspecific neurological symptoms were included. Both cMRI and 3D SI were acquired on single-vendor 1.5 T and 3 T scanners with slice thicknesses of 1.0–1.7 mm. Two experienced neuroradiologists and one fellow independently evaluated matched scans using a 0–100 scale. Assessed parameters included signal-to-noise ratio (SNR), gray–white matter contrast, artifacts, motion robustness, and confidence in detecting perivascular spaces, white matter lesions, and subtle pathology. Interrater agreement was measured using Krippendorff’s alpha and ICC2. Multiple linear regression analyzed associations between image quality ratings and imaging method. **Results**: Images of 31 patients were analyzed. Three-dimensional SI demonstrated sufficient-to-good overall image quality and high robustness to motion. Cortical-surface-to-cerebrospinal-fluid contrast on FLAIR was rated lower for 3D SI than for cMRI. False-positive lesion detection occurred more frequently on 3D SI FLAIR, particularly among experienced readers. cMRI achieved significantly higher T1-weighted SNR than 3D SI (8.76 points, *p* < 0.001). Experienced readers consistently rated SNR and tissue contrast higher than the fellow. Vascular signal range was broader on 3D SI, reducing sensitivity to vascular abnormalities. **Conclusions**: Three-dimensional synthetic MRI provides clinically usable image quality and fulfills its primary diagnostic purpose, offering advantages in acquisition efficiency and robustness to motion. Nevertheless, limitations in cortical contrast, vascular signal characterization, and reader-dependent interpretive variability constrain its reliability for subtle or detail-critical findings.

## Simple Summary

This study evaluates whether modern three-dimensional synthetic MRI (3D SI) provides reliable diagnostic information compared with conventional MRI (cMRI) in routine neuroradiology. Using matched datasets and multiple blinded readers, it assesses image quality, diagnostic confidence, and detection of key brain findings. Results show that 3D SI delivers usable, motion-robust images, though some anatomical features appear differently due to its technical principles, requiring readers to learn their distinct “synthetic” appearance. While cMRI offers higher confidence for subtle abnormalities, this advantage is tied to reader experience, which affects consistency across both techniques. The study outlines strengths, limitations, and practical guidance for integrating synthetic imaging into clinical workflows.

## 1. Introduction

Magnetic resonance imaging (MRI) is an established technique for diagnosing cranial pathologies. However, it involves the serial acquisition of multiple contrast-weighted sequences, which is a time-consuming process. Reducing scan duration without compromising diagnostic information could improve scanner availability, enhance patient comfort, increase throughput, and offer economic advantages.

Among several innovations aimed at accelerating MRI, synthetic imaging (SI) has emerged as a promising technique. The basis for SI is a quantitative MRI technique: the Quantification of Relaxation times and Proton density by Multi-Echo Acquisition of a Saturation-recovery using Turbo spin-Echo Readout (QRAPMASTER). In summary, the technique measures multiple tissue properties, primarily T1 and T2 relaxation time and proton density. From this, it generates multiple contrast-weighted images from a single acquisition. This enables the reconstruction of various image contrasts—such as T1-weighted (T1w), T2-weighted (T2w), and FLAIR-weighted images—without the need for separate acquisitions. In contrast, conventional MRI (cMRI) typically acquires one contrast weighting per sequence, requiring multiple acquisitions to achieve a full diagnostic set.

In clinical practice, radiologists still routinely make diagnoses based on conventionally weighted contrast images rather than using quantitative maps such as relaxation maps. Ideally, to the human eye, synthetic images should be similar to those conventionally acquired with an MRI scanner, both in terms of tissue information accuracy and artifacts. For economic reasons, it had been favorable to replace cMRI sequences with two-dimensional (2D) SI at our institution. But 2D SI did not allow for reconstruction in multiple planes, which is a key instrument in magnetic resonance (MR) diagnostics. With three-dimensional (3D) SI available, we are preparing to replace 2D SI with 3D SI.

However, while the image quality of 2D SI was known to us and has been evaluated in multiple prior studies [1,2,3,4,5], there is still limited literature assessing the quality of 3D SI. The Fujita research group was the first to report on the clinical evaluation of 3D SI. Their studies included measurements of cortical thickness and volumetric analyses, demonstrating that 3D SI can reliably measure cortical thickness and subcortical volumes in the human brain, with some regional exceptions [6]. In addition, they evaluated the utility of 3D SI in 24 patients with multiple sclerosis (MS) by comparing diagnostic image quality and lesion volumetry. They concluded that 3D SI could serve as an alternative to conventional MR imaging for the evaluation of MS [7]. Hagiwara et al. [8] evaluated whether 3D SI was suitable for detecting MS plaques and concluded that synthetic double inversion recovery images were superior to conventional double inversion recovery images in this regard.

Heo et al. [9] assessed the diagnostic image quality of 3D SI in clinical practice. Two neuroradiologists reviewed imaging data from 47 patients, evaluating overall image quality, anatomical delineation, and the presence of artifacts. Their study also incorporated compressed sensing (CS). They concluded that, in its current state, 3D SI cannot fully replace conventional brain MRI in routine clinical practice but noted that 3D SI can achieve scan-time reduction when combined with CS and parallel imaging.

Based on these findings, the present study aimed to assess the overall image quality of 3D SI and to evaluate its feasibility for integration into a clinical environment. Furthermore, we sought to determine which conventional imaging-based diagnostic knowledge can be directly applied to 3D synthetic imaging and which aspects cannot. In contrast to previous studies, no additional compressed sensing techniques were employed in this work. While recent reviews have summarized the clinical characteristics and limitations of predominantly 2D synthetic MRI techniques [5], systematic reader-based evaluations of 3D synthetic MRI-derived image quality and diagnostic confidence remain limited.

## 2. Materials and Methods

### 2.1. Study Design

All MRI examinations were performed at Lucerne Cantonal Hospital, Central Switzerland, as part of routine clinical care. Scans of patients who underwent both 3D SI and cMRI between 1 March 2020 and 31 August 2022 were evaluated. The initial indication for the 3D SI was the quantification of brain volume and myelin (not part of this evaluation), and the evaluation of the weighted images was performed separately and retrospectively.

### 2.2. Image Acquisition

MRI examinations were conducted using two systems from the same manufacturer: a 1.5 T Siemens Magnetom Aera and a 3 T Siemens Magnetom Vida fit (Siemens Healthcare, Erlangen, Germany), each equipped with a 20-channel head coil.

The 3D SI protocol was based on a quantitative MRI sequence known as QALAS (Quantification using an interleaved Look–Locker acquisition sequence with T2 preparation). The technique is described in detail by Warntjes et al. [10]. This non-triggered, 3D spoiled gradient-echo sequence captures five image readouts with varying contrasts within a single acquisition cycle, approximately one per second. The sequence begins with a T2 preparation pulse, followed by an inversion pulse and four subsequent readouts. T1 relaxation times are fitted using the four post-inversion acquisitions, while T2 relaxation times are estimated from signal intensity changes between the first and fifth acquisitions [11,12,13].

Three-dimensional SI quantitative data were post-processed with SyMRI software (version 22Q3; v0.45.38, SyntheticMR AB), and reconstructed T1w, T2w, and FLAIR imaging data were archived on a PACS (Picture Archiving and Communication System) along with cMRI Data.

Three-dimensional SI images were reconstructed at the following slice thicknesses: 1.0 mm, 1.2 mm, 1.33 mm, 1.5 mm, and 1.7 mm. The 1.0 mm, 1.2 mm, and 1.33 mm datasets were acquired exclusively on the 3 Tesla scanner, whereas the 1.5 mm and 1.7 mm datasets were obtained on both the 1.5 Tesla and 3 Tesla systems. Acquisition parameters for both 3D SI and cMRI are summarized in Table S1a,b in the Supplementary Materials.

### 2.3. Evaluation of Imaging Quality

To assess the image quality and diagnostic characteristics of 3D SI, three readers, a neuroradiology resident R1 (2 years) and two neuroradiologists—R2 (15 years of experience) and R3 (14 years)—independently reviewed the T1w, T2w, and FLAIR images.

All readers were blinded to the patients’ clinical histories and first evaluated 3D SI and cMRI with a minimum interval of 2 weeks. Image windowing parameters were initially set according to predefined values (Table S2 in the Supplementary Materials) but were allowed to be adjusted manually by the readers for optimal visualization if deemed necessary.

### 2.4. Qualitative Image Assessment Parameters

The three readers evaluated the following imaging characteristics on 3D SI-weighted images: signal to noise and contrast, capacity of signal suppression of cerebral and spinal fluid (CSF), pulsation effects in the basilar artery, signal of the venous sinus, CSF pulsation in prepontine cistern, overall motion artifacts, cortex versus white matter contrast, discriminability of lentiform nucleus and sulci, and identifiability of white matter lesions (Table 1). The selection of evaluated parameters was informed by previously reported qualitative assessment frameworks in synthetic MRI literature (e.g., Sharma et al. [5]); however, the scoring scales, operational definitions, and application to 3D synthetic MRI datasets were specifically developed for the present reader-based comparative study.

### 2.5. Reader Training and Rating Procedure

All three readers rated each imaging parameter. Prior to the main assessment, a calibration session was conducted using three representative cases (excluded from the final analysis) to familiarize the readers with the scoring methodology.

Each reader evaluated the images on a continuous scale from 0 to 100 to evaluate imaging characteristics as listed in Table 1. To promote consistency in scoring, a structured rating framework was applied: readers first selected a general range using a mental mind-map divided into four quartiles (for mind map quartiles, see Table 1). These served as a preliminary guide before assigning a final, precise score within the selected interval. The quartiles were not documented individually but served as internal reference points.

Global cortical atrophy (GCA) was rated on the Pasquier scale 0–3 [14].

### 2.6. Evaluation of Diagnostic Confidence

Diagnostic confidence in identifying perivascular spaces and remnant cysts was assessed in anatomically characteristic locations, namely the basal ganglia, anterior commissure, and hippocampus. Confidence in lesion detection within white matter was also evaluated, with a lesion defined as a focus of hyperintensity ≥3 mm in diameter. For this assessment, conventional FLAIR, T1w, and T2w images were reviewed in parallel.

### 2.7. Statistical Analysis

The analyses were mainly descriptive. To illustrate key findings, we generated boxplots and scatterplots comparing the various imaging parameters assessed by the three independent readers (R1, R2, and R3) for both cMRI and 3D SI. These chosen visualizations highlighted differences in image quality attributes, contrast perception, and diagnostic confidence across modalities and among raters. The visualizations were created with the ggplot2 package (version 3.5.1) [15]. We computed the interobserver agreement using Krippendorff’s alpha (icr package, version 0.6.4) and the intraclass correlation (ICC2s; psych package [16], version 2.4.1). Agreement categories were defined as follows: α = 1—*perfect agreement*, α ≥ 0.80—*satisfactory level of agreement*, 0.67 ≤ α < 0.79—*moderate agreement*, and α < 0.67—*poor agreement* [17,18]. Agreement categories (Krippendorff’s α) are presented in italics to distinguish them from overlapping terminology used in the mind-map quartiles, which pertain to parameter scaling.

Linear or logistic regression models were developed to assess the association between each imaging parameter (outcome) and the methods (cMRI vs. 3D SI; finalfit package; version 1.0.7) [19]. Readers (R1, R2, R3), scan magnetic field strength (1.5 T, 3 T), and thickness of image slices (1mm, 1.2 mm, 1.3 mm, 1.5 mm, 1.7 mm) were adjusted for in the models. The regression models for the following parameters are presented below:

SNR on FLAIR, contrast of CSF in sulci against cortex in FLAIR, contrast of lesions in white matter against adjacent tissue, and head-motion-related degradation on image quality. In the, Section 3 emphasis was placed on medians, interquartile range (IQR), effect size, and confidence intervals (CIs) over statistical significance. Descriptive and inferential statistical analyses were performed using statistical software R (version 4.4.1) [20].

## 3. Results

### 3.1. Patient and Scan Characteristics

A total of 31 patients who underwent 3D SI on two different MRI scanners (1.5 Tesla and 3 Tesla) and had corresponding conventional 3D FLAIR, T1w, and T2w imaging available for comparison were included in the analysis. The 3D SI acquisitions were performed with the following slice thicknesses: 1.0 mm (*n* = 4), 1.2 mm (*n* = 1), 1.33 mm (*n* = 12), 1.5 mm (*n* = 3), and 1.7 mm (*n* = 11).

The clinical indications for imaging were suspected intracranial tumors (*n* = 4), nonspecific neurological symptoms (*n* = 14), and evaluation for possible demyelinating white matter lesions (*n* = 13). The median age of the patient cohort was 48 years (interquartile range (IQR): 33–59.5 years).

In 16 of the 31 patients, images were obtained after administration of contrast medium: conventional FLAIR sequences in 13 patients and 3D SI in 3 patients.

### 3.2. Image Quality Assessment

**Figure 1:**
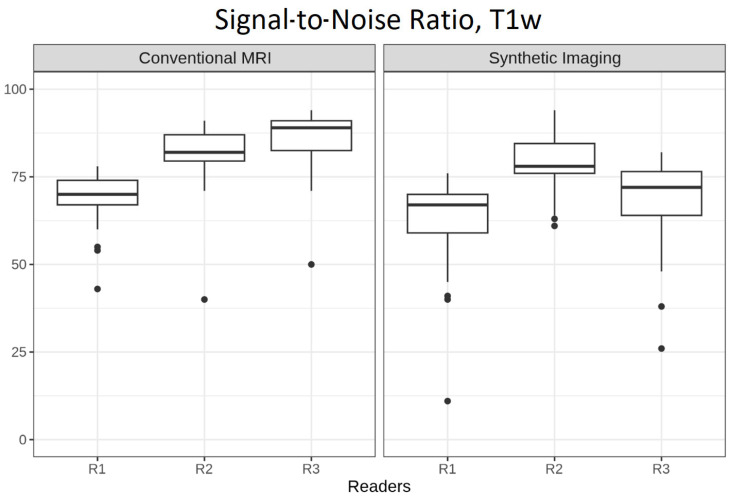
Boxplots comparing the variable “Signal-to-Noise Ratio” for T1w images across the three readers (R1, R2, and R3) for both conventional and synthetic MRI. The *y*-axis shows ratings on a 0–100 scale for the image characteristic.

Figure 1 presents boxplots comparing the variable SNR for T1w images across the three readers (R1, R2, and R3) for both cMRI and 3D SI. For cMRI, the SNR ratings were generally higher than for 3D SI. R3 reported the highest scores, with a median (IQR) of 89 (82.5–91). R2 demonstrated a slightly lower median (IQR) rating of 82 (79.5–87), and R1 achieved a rating of 70 (67–74). For 3D SI, R2 reported the highest scores with a median (IQR) of 78 (76–84.5).

Interrater agreement, as measured by Krippendorff’s alpha, was α = 0.277 for cMRI and α = 0.168 for 3D SI.

**Figure 2:**
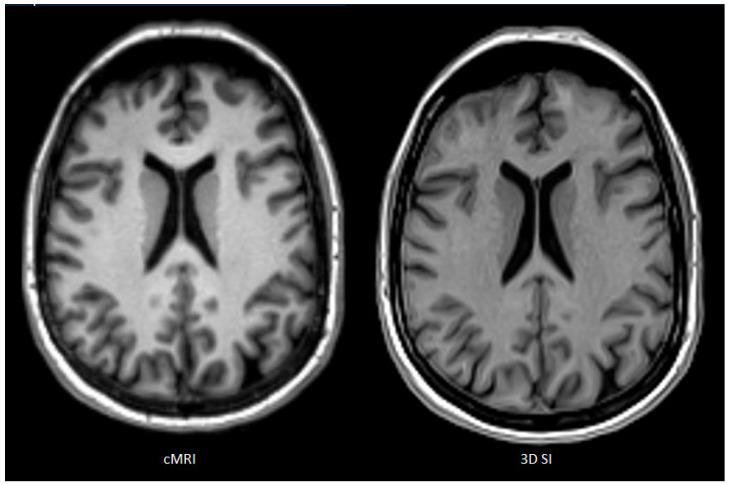
Slice of a single patient’s T1w images for each cMRI and 3D SI as an example for perceived signal to noise (cMRI: R1, 87; R2, 91; R3, 76; and 3D SI: R1, 68; R2, 76; R3, 62).

Figure 2 shows a slice of a single patient’s T1w images for each cMRI (left) and 3D SI (right) as an example for perceived SNR ratings: cMRI: R1, 87; R2, 91; R3, 76; and 3D SI: R1, 68; R2, 76; R3, 62.

In addition to the boxplot analysis, Table 2 provides a quantitative comparison of the SNR ratings (0–100 scale; labeled “Signal-to-Noise Ratio”) between T1w cMRI (reference) and 3D SI, based on multiple linear regressions. Independently of all readers, 3D SI images were rated on average (95% confidence interval (CI)) 8.76 (−11.26 to −6.27, *p* < 0.001) points lower than their conventional counterparts. Interrater comparison using R1 as the reference demonstrated that R2 and R3 assigned SNR ratings that were 14.63 (11.57 to 17.69, *p* < 0.001) and 11.40 (8.35 to 14.46, *p* < 0.001) points higher, respectively.

To account for potential confounding variables, slice thickness and magnetic field strength were included as covariates in the adjusted linear regression model. Although exploratory in nature, the analysis revealed that imaging performed at 3T was associated with a 6.73-point advantage (0.89 to 12.58, *p* = 0.012) in SNR rating over 1.5 Tesla imaging. In contrast, slice thickness did not demonstrate a consistent trend. Estimates varied widely across thicknesses, with no clear preference for thinner or thicker slices.

### 3.3. Evaluation of Signal to Noise and Contrast Parameters Across Modalities

**Figure 3:**
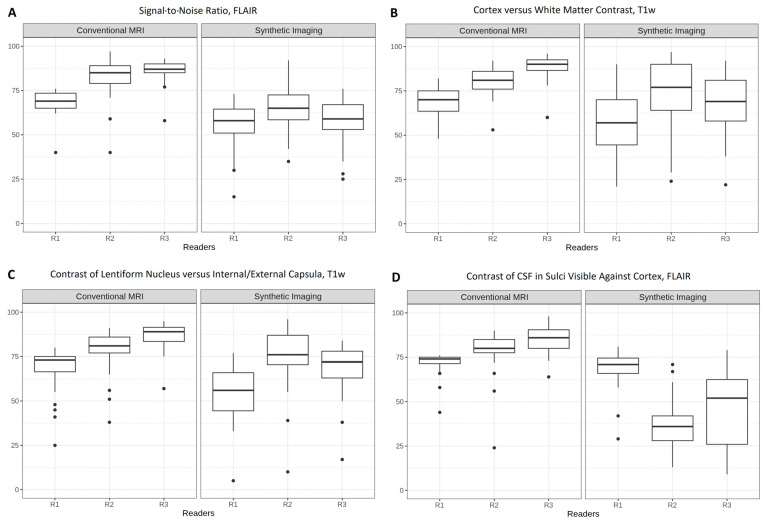
Boxplots of reader ratings for conventional MRI and 3D synthetic imaging across four key image characteristics: “Signal-to-Noise Ratio, FLAIR” (**A**), “Cortex versus White Matter Contrast, T1w” (**B**), “Contrast of Lentiform Nucleus versus Internal/External Capsula, T1w” (**C**), and “Contrast of CSF in Sulci Visible Against Cortex, FLAIR” (**D**). FLAIR, fluid-attenuated inversion recovery. CSF, cerebrospinal fluid. The *y*-axis shows ratings on a 0–100 scale for the image characteristic. Krippendorff’s alpha cMRI vs. 3D SI: A, 0.177 vs. 0.194; B, −0.053 vs. 0.588; C, 0.343 vs. 0.579; D, 0.239 vs. 0.064.

Figure 3 presents boxplots comparing reader ratings for cMRI and 3D SI across four key image characteristics: SNR on FLAIR (A), contrast between cortex and white matter on T1w images (B), contrast between the lentiform nucleus and internal/external capsule on T1w images (C), and contrast of cerebrospinal fluid (CSF) in sulci against the cortex on FLAIR (D). For SNR (A), cortex versus white matter contrast (B), and contrast of lentiform nucleus versus internal/external capsula (C), ratings remained broadly consistent with previously observed patterns in SNR in T1w evaluation (Figure 1). On the mind-map quartile range, R2 and R3 consistently rated cMRI as excellent, while R1 mostly rated cMRI as good. Three-dimensional SI was rated mostly as good to excellent by R2 and R3, whereas R1 gave lower ratings, mostly ranging from good to sufficient.

Contrast agreement between cortex and white matter in T1w imaging (B) was *poor* for both cMRI (Krippendorff’s alpha = −0.053) and 3D SI (Krippendorff’s alpha = 0.588). Contrast between the lentiform nucleus and the internal/external capsule (C) showed greater, but still *poor* interrater agreement for 3D SI (Krippendorff’s alpha = 0.579) compared to cMRI (Krippendorff’s alpha = 0.343).

Ratings for the contrast of CSF in sulci against cortex in FLAIR (D) in cMRI also followed a similar pattern to SNR in T1w evaluation, whereas the ratings in the 3D SI did not. Medians (IQRs) for 3D SI were R1, 71 (66–74.5); R2, 36 (28–42); and R3, 52 (26–62.5). For cMRI, they were R1, 74 (71.5–75); R2, 80 (77.5–85), and R3, 86 (90–90.5).

### 3.4. Assessment of Signal Properties in Moving Structures and Fluid Spaces

**Figure 4:**
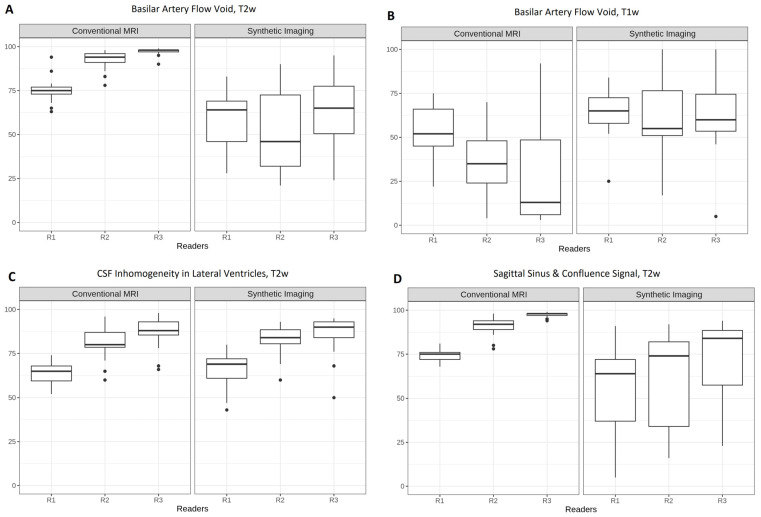
Boxplots of reader ratings of images on signal of basilar artery in T2w (**A**), basilar artery T1w (**B**), CSF (**C**), and venous sinuses T2w (**D**). Krippendorff’s alpha cMRI vs. 3D SI: A, −0.419 vs. 0.191; B, 0.056 vs. 0.545; C, −0.165 vs. −0.170; D, −0.420 vs. 0.570. The *y*-axis shows ratings on a 0–100 scale for the image characteristic.

Figure 4 summarizes reader ratings of signal characteristics in anatomically dynamic or fluid-filled regions, particularly focusing on flow-related signal voids and CSF appearance. In cMRI, the hypointense signal of the basilar artery (Figure 4A) and venous sinuses (Figure 4D)—traditionally referred to as “flow void” in T2w cMRI imaging [21]—was evaluated as black or dark gray by all readers (A, median (IQR): R1, 75 (73–77); R2, 94 (91–96); R3, 98 (97–98); and D, median (IQR): R1, 75 (72–76); R2, 92 (89–94); R3, 98 (97–98)). Compared to cMRI, the signal intensity in 3D SI showed lower medians and much broader IQRs (A, median (IQR): R1, 64 (46–69); R2, 46 (32–72.5); R3, 65 (50.5–77.5); and D median (IQR): R1, 64 (37–72); R2, 74 (34–82); R3, 84 (57.5–88.5)).

Median values of T1w signals in the basilar artery (Figure 4B) were closer to each other for 3D SI (B, median (IQR): R1, 65 (58–72.5); R2, 55 (51–76.5); R3, 60 (53.5–74.5)) than for cMRI (B, median (IQR): R1, 52 (45–66); R2, 35 (24–48); R3, 13 (6–48.5)). They also di-verged notably from the signal characteristics observed on cMRI.

In contrast, CSF signal within the ventricles (Figure 4C) demonstrated *poor* interreader agreement for both cMRI and 3D SI (Krippendorff’s alpha: −0.165 and −0.170, respectively). However, intrareader assessments were consistent across modalities, with each reader assigning similar homogeneity scores to cMRI and 3D SI. Specifically, reader 1 rated CSF signal homogeneity as good for both modalities, while readers 2 and 3 rated it as excellent.

### 3.5. Contrast of Cerebral Fluid in Sulci Against Cortex

**Figure 5:**
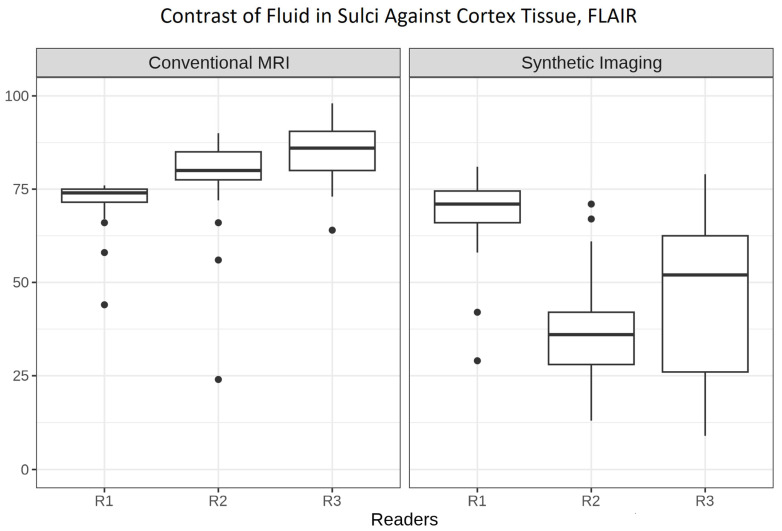
Boxplots comparing the variable “Contrast of Fluid in Sulci Against Cortex Tissue” for FLAIR images across readers R1–R3 for both conventional and synthetic MRI. The *y*-axis shows ratings on a 0–100 scale for the image characteristic.

Figure 5 presents boxplots comparing the variable “Contrast of Fluid in Sulci Against Cortex Tissue” for FLAIR images across readers for both cMRI and 3D SI. Signal of CSF in the sulci shows good-to-excellent contrast against cortex of the brain in cMRI (median (IQR): R1, 74 (71.5–75); R2, 80 (77.5–85); R3, 86 (80–90.5). Signal of CSF in the sulci shows only sufficient-to-good contrast against cortex of the brain in 3D SI (median (IQR): R1, 71 (66–74.5); R2, 36 (28–42); R3 52 (26–62.5)).

Interrater agreement, as measured by Krippendorff’s alpha, was α = 0.239 for cMRI and α = 0.064 for 3D SI.

Adjusted linear regression estimates showed lower scores for “Contrast of Fluid in Sulci Against Cortex Tissue” in 3D SI compared to cMRI (−27.75 (−31.99–23.51, *p* < 0.001)).

**Figure 6:**
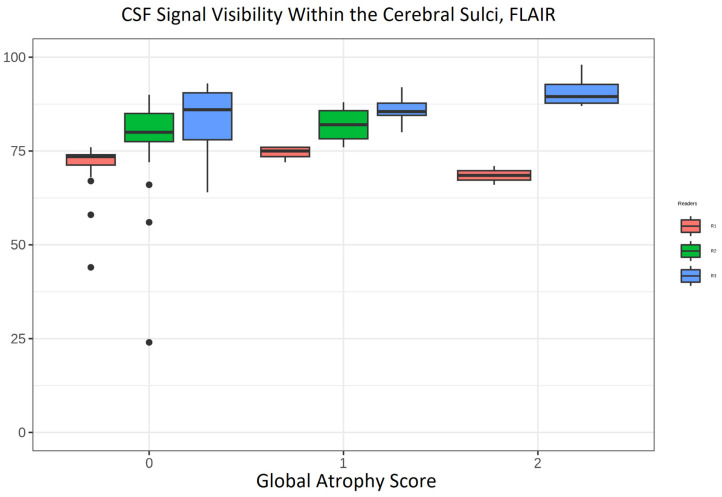
Boxplots comparing the distribution between the variable “CSF Signal Visibility Within the Cerebral Sulci” FLAIR images across the three readers for 3D SI and the global atrophy score on the *x*-axis for R1 to R3. The *y*-axis shows ratings on a 0–100 scale for the image characteristic.

In cerebral atrophy, the cortical sulci widen. Sulcal width may therefore represent a parameter influencing the effectiveness of CSF suppression within the sulci on MRI. Because readers 2 and 3 rated CSF suppression as sufficient to good—see Figure 5 (reader 2: 36 (28–42); reader 3: 52 (26–62.5)—we investigated whether sulcal widening could act as a confounding factor. To demonstrate a possible association between the width of the sulci and the signal of CSF, we used Figure 6 to demonstrate this relationship (rated by GCA on cMRI on the *x*-axis) and CSF signal on 3D SI FLAIR.

Median values for CSF signal visibility show an upward trend with increasing GCA scores for R3 (medians, (IQRs)): GCA 0 (31 (18.5–55.5)), GCA 1 (59 (51.75–64.75)), GCA 2 (74 (68.5–77.5)); but not for R1 or R2.

### 3.6. Lesion Visibility and Recognition Confidence

**Figure 7:**
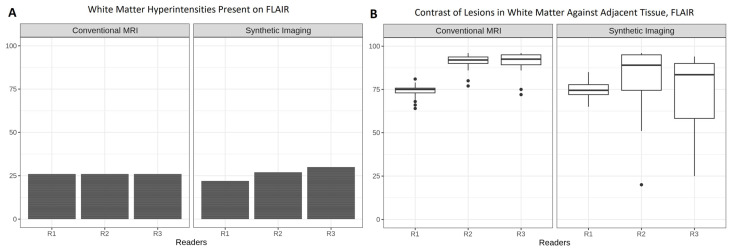
Bar plots of the variable “White Matter Hyperintensities Present on FLAIR” showing the number of cases identified by the readers (**A**) and their corresponding boxplots showing the confidence that each reader felt in recognizing their presence (**B**). The *y*-axis shows ratings on a 0–100 scale for the image characteristic.

Figure 7A demonstrates detection of white matter hyperintensities present in FLAIR imaging. All three readers identified white matter hyperintensities in the same 26 of 31 patients in cMRI (Krippendorff’s Alpha of 1.000). In contrast, ratings for 3D SI showed *poor* agreement (Krippendorff’s Alpha of 0.335). Except for R1, the readers detected more hyperintensities compared to cMRI.

Confidence in distinguishing true hyperintensities (lesions) from noise artifacts was assessed as perceived contrast (Figure 7B). The experienced readers (R2 and R3) demonstrated higher median scores than the less experienced reader (R1) for contrast for cMRI versus 3D SI (cMRI, median (IQR): R1,75 (73–75.75); R2, 92 (90–93.75); R3, 92.5 (89.25–95); 3D SI, median (IQR): R1, 74.5 (72–77.75); R2, 89 (74.5–95); R3, 83.5 (58.25–90)). However, their IQRs in 3D SI were wider.

Adjusted linear regression estimates showed lower scores for contrast of lesions in 3D SI (−7.74 (−11.16 to −4.31, *p* < 0.001)) compared to cMRI.

Despite the higher medians for the perceived contrast of the lesions against their white matter background, surprisingly, both experienced readers R2 and R3 reported hyperintensities in 3D SI in the same two patients, but not in cMRI (false positives). R3 reported false-positive hyperintensities in two additional patients. All false-positive hyperintensities appeared at cortical CSF boundaries and along sulcal surfaces.

### 3.7. Signal-to-Noise Ratio in Relation to Motion Robustness

**Figure 8:**
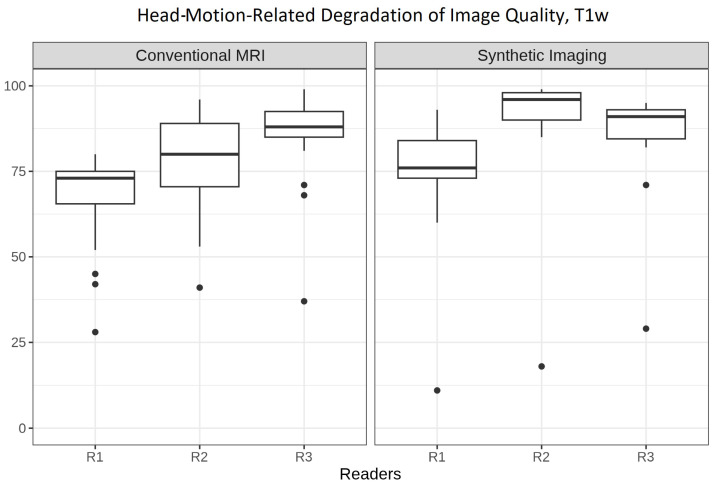
Boxplots of the image quality degradation from patient motion across conventional MRI and 3D synthetic imaging. The *y*-axis shows ratings on a 0–100 scale for the image characteristic.

Figure 8 summarizes reader impressions of head-motion-related degradation on image quality, regarding T1w imaging. Three-dimensional SI and cMRI were, independently of each other, subject to motion artifacts. Both cMRI and 3D SI demonstrated good-to-excellent overall impressions.

However, adjusted linear regression estimates indicated that 3D SI received motion robustness ratings 6.97 points higher than cMRI (4.73 to 9.20, *p* < 0.001). Krippendorff’s alpha reached 0.665 for 3D SI, but only 0.393 for cMRI.

**Figure 9:**
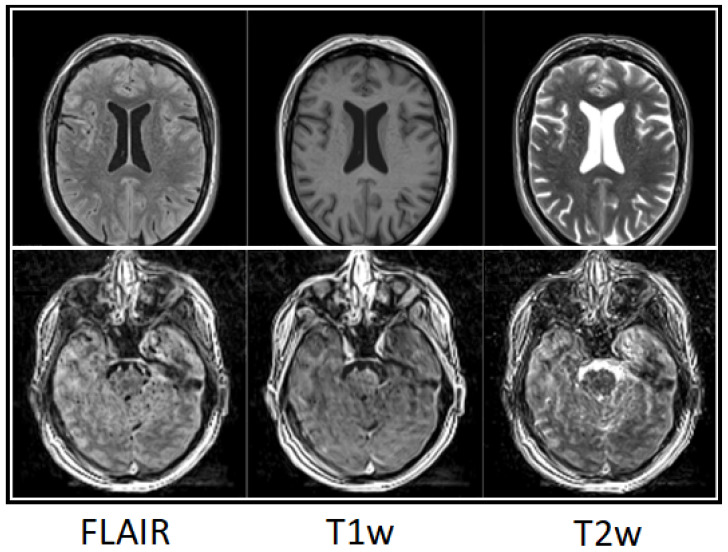
Three-dimensional SI examples of perceived motion on derived image weightings (from left, FLAIR, T1w, T2w) rated on T1-weighted 3D SI. Patient images in upper row ratings (cMRI) R1, R2, R3: 92, 95, 92, and lower row 11, 18, 29, respectively.

Figure 9 demonstrates an impression of perceived head motion for two different patients. Mind-map scores for the derived image weightings FLAIR, T1w, and T2w for the patient in the upper row showed excellent quality with no relevant degradation (upper-row ratings (cMRI) R1, R2, R3: 92, 95, 92). Mind-map scores for the patient in the lower row were poor to sufficient (lower-row ratings (3D SI) R1, R2, R3: 11, 18, 29).

**Figure 10:**
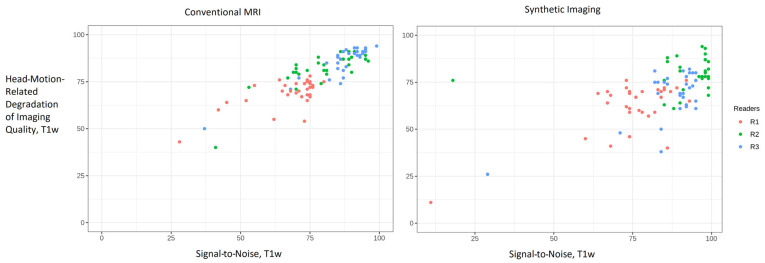
Scatterplot comparing cMRI and 3D SI of signal to noise in T1w (*x*-axis) versus “Head-Motion-Related Degradation of Image Quality, T1w” (*y*-axis) in T1w. The dots represent the evaluation by reader 1, 2, and 3 (color-coded).

Figure 10 explores the relationship between readability despite motion artifacts (*x*-axis) and perceived image quality (*y*-axis) for SNR in T1w images.

For cMRI and 3D SI data points gathered in the upper-right quadrant, without the color coding of the readers, there would appear to be a correlation between motion and image quality. However, when separately shown for each reader, as indicated by the different colors, it reveals the clustering of the data by reader. For readability despite motion artifacts, the medians (IQRs) are as follows: cMRI motion—R1, 73 (65.5–75); R2, 80 (70.5–89); R3, 88 (85–92.5); 3D SI motion—R1, 76 (73–84); R2, 96 (90–98); R3, 91 (84.5–3). Values for SNR in T1w are given in Figure 1.

### 3.8. Artifacts

**Figure 11:**
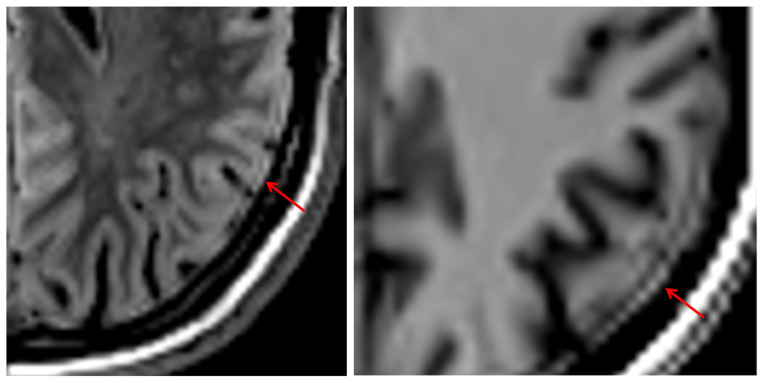
Hyperintense rim at the outer border of the cortex and dura in T1w 3D SI.

Of note, we found a thin hyperintense rim without echo contour on all 3D SI images, which delineated the surface of the brain. Figure 11 demonstrates this on a 3D SI FLAIR and T1w image. It is readily recognizable as an artifact, because it crosses anatomic borders.

In 3 of the 31 cases, contrast mediums were administered prior to acquisition of the QALAS sequence and in 13 of 31 patients prior to the acquisition of the conventional FLAIR sequences. As expected, ratings for 3D SI T1w images in these cases showed hyperintense signals in the basilar artery and venous sinuses, consistent with contrast enhancement of central vessels for these three subjects. The effect of the contrast medium was obvious to the readers for both 3D SI and cMRI, because the choroid plexus was visibly enhanced. However, due to the small number of subjects (3/31 for 3D SI), we did not statistically evaluate the confounding effects on the other evaluated parameters.

## 4. Discussion

This study aimed to evaluate the imaging characteristics of 3D SI, with a focus on clinically relevant tissue contrasts and anatomical details, such as image noise and tissue contrast. SNR is vital in MRI because it underpins image clarity, measurement accuracy, contrast detection, scan efficiency, and reproducibility. Contrast ensures that tissues and pathologies are distinguishable. Without adequate SNR and contrast, both visual interpretation and quantitative evaluation of clinical parameters become unreliable.

Three readers, two experienced neuroradiologists and one neuroradiology resident, compared and assessed brain cMRI and 3D SI images acquired in a clinical setting. They evaluated them on a 0–100 scale to enable fine-grained evaluation of the parameters. Overall, reader-specific rating patterns were consistent across the evaluated image characteristics. Notably, the experienced readers demonstrated higher interrater agreement and generally provided more favorable ratings for SNR and gray–white matter contrast across both cMRI and 3D SI. In contrast, the less experienced reader tended to assign lower scores, indicating reduced satisfaction with image quality for both techniques.

Three-dimensional SI showed sufficient-to-good performance in terms of SNR and contrast, except for sulcal contrast in FLAIR. Although not achieving excellence, the quality was clinically sufficient.

However, further optimization is required to ensure consistent, high-level performance. When distinguishing meningitis from normal brain surface, CSF signal suppression in FLAIR imaging is of critical importance to rule out diseases which manifest on the meninges and the subarachnoid space [22]. In our ratings, CSF signal in 3D SI was rated more poorly because its behavior deviated from what is expected in conventional imaging, and in fact its performance was only sufficient to good.

We had suspected that on 3D SI images, the degree of sulcal widening could possibly influence the CSF signal. We suspected that with increasing width of sulci, the CSF signal could begin to approximate the expected hypointense appearance in FLAIR. Atrophy, as measured by GCA score, would be a surrogate marker for width of sulci. However, as we related the GCA scores to the CSF signal, the readers did not consistently confirm this impression. Median values for CSF signal visibility demonstrated an upward trend with increasing GCA scores for reader 3 only, whereas no such trend was observed for readers 1 or 2. Notably, the ratings of the inexperienced reader were not aligned with those of either experienced reader. This pattern suggests that the observed variability is more likely attributable to insufficient prior training on the dataset rather than to differences in reader experience per se.

This incomplete CSF suppression has previously been described [11]. It is presumed to arise from partial volume effects involving brain tissue and CSF. Importantly, sulcal artifacts in FLAIR 3D SI should not be misinterpreted as evidence of meningitis, nor should they be used to exclude its presence.

No consistent explorative patterns across parameters were observed with respect to the covariates’ field strength and slice thickness for 3D SI. Since variations in slice thickness did not significantly affect the perceived quality of signal, noise, or contrast on 3D SI, our results indicate that imaging with slices in the range of 1–1.7 mm can be achieved without compromising overall image quality. This represents an important advantage, as the resulting higher spatial resolution facilitates the visualization of small anatomical structures that were previously limited in conventional 2D spin-echo acquisitions.

When multiple weightings are derived from a single acquisition, as in 3D SI, patient motion during the scan uniformly affects all derived contrasts. Given this, we aimed to investigate the robustness of 3D SI under typical clinical conditions. Our results concerning motion during acquisition of imaging (Figure 8) showed that 3D SI maintains sufficient image readability even in the presence of motion.

The inconsistent administration of gadolinium-based contrast across patients and across modalities constitutes a source of bias in this study. In 13 of 31 patients, cMRI FLAIR was acquired after contrast administration, whereas post-contrast acquisition occurred in only 3 patients for 3D SI. This imbalance introduces confounding because post-contrast signal behavior is not equivalent across tissues and sequences: enhancement of extra-axial structures (e.g., choroid plexus and dura) was visually evident to readers, potentially unblinding scan conditions and shifting subjective ratings. Moreover, contrast can alter conspicuity of leptomeningeal and vascular structures on FLAIR and may influence sulcal signal patterns, which are central to our evaluation of CSF suppression and the risk of meningitis overcalling. While major intra-axial contrast changes are not typically expected for conventional FLAIR in many indications, even subtle differences in timing, dose, and sequence-specific sensitivity may affect perceived gray–white matter contrast, lesion conspicuity, and artifact interpretation [23]. For 3D SI, where multiple contrasts are synthetically derived and sequence behavior is less familiar, the direction and magnitude of any post-contrast effects are less predictable. Consequently, part of the observed interreader variability and technique-specific impressions may reflect heterogeneous contrast timing rather than intrinsic properties of 3D SI versus cMRI.

In the multiple linear regression assessing the relationship between SNR and T1w imaging methods (cMRI and 3D SI; Table 2), readers R2 and R3 assigned ratings that were, on average, 14.63 and 11.40 points higher than the less experienced reader, respectively. This illustrates an important degree of subjectivity in image quality perception among radiologists despite the calibration session. However, such findings are consistent with previous reports evaluating subjective MR image quality, where Kappa results tend to be low due to the inherent subjectivity of visual scoring and the chosen range of rating scales. Therefore, while Kappa results provide a quantitative measure of agreement, they may not fully capture the consistency in qualitative judgment across raters [24,25]. Important to note is that it also suggests that variability between readers may exceed the impact of actual image quality improvements. Interrater reliability was notably stronger for cMRI, where agreement ratings suggested a well-established, shared expectation of image contrast among radiologists. In contrast, 3D SI appeared more reader-dependent, with increased divergence in interpretation, particularly in motion-compromised images or when assessing nuanced contrast boundaries. This highlights a potential vulnerability of 3D SI to reader subjectivity in detail-critical evaluations. This could reflect unfamiliarity with the image impressions, as well as the absence of established perceptual “engrams” that are typically developed through repeated exposure to cMRI images in routine clinical practice. Radiologists build diagnostic confidence not only from objective signal properties but also from accumulated experiential patterns—mental templates formed through consistent interpretation of standard imaging appearances. Three-dimensional SI, being relatively novel, may lack this embedded familiarity, leading to greater variability in interpretation and potentially more cautious or conservative ratings, especially in complex or borderline cases.

From an implementation perspective, these findings argue against an immediate, unstructured replacement of conventional sequences by 3D SI in routine reporting. Instead, a structured training pathway should precede broader clinical rollout. Such a pathway should explicitly teach the modality-specific appearance of normal anatomy, expected contrast behavior, and recurrent 3D SI artifacts that are prone to misinterpretation, including incomplete CSF suppression in sulci on FLAIR, the cortical hyperintense rim, and altered depiction of flow voids. Importantly, our results suggest that this need is not limited to trainees: even experienced readers may require recalibration of diagnostic heuristics when transitioning from established 2D/3D cMRI appearances to the novel contrast impressions of 3D SI.

A practical approach would include an annotated reference atlas with representative normal and pathological cases, supervised case-based reading with immediate feedback and consensus review, and periodic calibration sessions with predefined acceptance criteria for key diagnostic tasks. During early adoption, quality assurance measures such as double reading, targeted correlation with conventional sequences in equivocal cases, and structured reporting checklists could mitigate the risk of false positives and false negatives while experience accrues. In parallel, future studies should prospectively quantify learning curves for 3D SI interpretation, for example, by tracking changes in interrater agreement, confidence ratings, and task-specific error patterns over sequential case exposure, and by defining competency thresholds required before independent clinical use.

On the one hand, experienced readers R2 and R3 tended to rate scores similarly and demonstrated a lower threshold for deeming images diagnostically acceptable than the reader in training, which may reflect greater familiarity with modality-specific artifacts and diagnostic workarounds. On the other hand, the experienced readers exhibited a wider IQR compared to reader R1, suggesting more interpretative uncertainty in the evaluation of presence of lesions in 3D SI FLAIR (Figure 7B). Despite being aware of diagnostic uncertainty, the experienced readers overcalled lesions on 3D SI. This observation supports prior findings by Fujita et al. [11], who noted a similar pattern of false positives associated with 3D SI, especially in cortical regions near CSF interfaces. It highlights the need for recalibration of diagnostic heuristics when interpreting novel imaging modalities, even among seasoned radiologists. It is important to note that our study did not specifically target typical MS lesions, nor did it compare dedicated imaging sequences such as double inversion recovery (DIR), which Hagiwara et al. [8] found to be superior in 3D SI (when optimized per patient) compared to cMRI. In our cohort, the overcalled lesions were not randomly distributed but appeared to cluster in regions where 3D SI is known to produce interface-related signal abnormalities, most notably at cortical CSF boundaries and along sulcal surfaces. This spatial predilection is consistent with prior observations that synthetic FLAIR and related reconstructions may show hyperintense rims and sulcal signal irregularities attributable to incomplete CSF suppression and partial-volume effects near the pial surface. Such patterns can mimic small juxtacortical plaques or superficial inflammatory changes and may therefore inflate false-positive white matter lesion counts, particularly when readers apply conventional cMRI heuristics to the novel 3D SI appearance.

One reason for overcalling may be increased sensitivity of the readers, implying that the experienced readers were more attuned to subtle image features resembling lesions. This may be attributable to their familiarity with lesion contrast on cMRI, leading them to interpret similar-appearing features on synthetic images as white matter lesions. However, synthetic images may limit the translation of this heightened sensitivity into a reliable reference standard, despite the high diagnostic confidence reported by the experienced readers. Another reason, on the other hand, may be reduced specificity, suggesting that the experienced readers interpreted image noise or artifacts as pathological findings, thereby increasing the number of false positives.

To systematically differentiate these artifacts from true pathology in daily practice, radiologists can apply a structured set of checks:

- Topography, as artifacts typically follow the cortical ribbon or conform to sulcal geometry rather than forming discrete ovoid lesions;
- Morphological stability across adjacent slices, as partial volume/interface effects often appear as thin curvilinear signal bands that fluctuate with sulcal caliber, whereas true lesions show more coherent 3D extent;
- Cross-contrast consistency, since true lesions should exhibit a plausible pattern across T2w/FLAIR/T1w-derived contrasts, while interface artifacts may be conspicuous on synthetic FLAIR but lack concordant appearance on other 3D SI weightings; and
- Correlation with conventional sequences or dedicated high-specificity contrasts when available, particularly in borderline juxtacortical findings.

In addition, establishing local reference libraries of typical 3D SI interface artifacts and incorporating targeted reader training can reduce “pattern completion” errors during early adoption, and structured reporting language can explicitly flag cortical–CSF interface hyperintensities as likely artifactual when they match these stereotyped features.

The hyperintense rim at the cortical surface, visible in all 3D SI weightings and previously reported by Blystad et al. [26] and described as “ringing artifact” by Fujita et al. [7], complicates confident detection of cortical, subdural, and dural lesions. While this artifact is recognizable and, once familiar, may not degrade interpretive quality, its presence must be acknowledged to avoid diagnostic missteps, such as misinterpreting dural thickening or subdural hematomas.

In routine reporting, conventional T2w/FLAIR imaging still provides a rapid, qualitative “sanity check” of vessel patency through expected flow voids in large arteries and venous sinuses. This becomes particularly relevant in emergency workflows and stroke-related imaging, where time-critical decisions may be informed by early suspicion of large-vessel occlusion or venous thrombosis before dedicated angiographic data are reviewed. In our experience, this diagnostic cue is diminished in 3D SI, where the broader synthetic signal range and reduced or absent flow-void effects can mask abnormal intravascular signal patterns. In real-world practice, this could translate into false reassurance on routine brain sequences, delayed escalation to targeted vascular imaging, or reduced confidence when triaging equivocal cases (e.g., basilar artery occlusion or dural sinus thrombosis). Consequently, 3D SI should not be relied upon as a substitute for flow-based patency assessment in acute neurovascular pathways. If 3D SI is incorporated into emergency MRI protocols, it should be accompanied by dedicated vascular sequences (e.g., TOF-MRA or contrast-enhanced MRA where appropriate) and interpreted with explicit awareness that “normal-appearing” intravascular signal on 3D SI does not exclude occlusion or thrombosis. For longitudinal morphometric imaging in neurodegenerative disease, highly standardized 3D T1-weighted sequences such as MPRAGE remain the reference standard due to their well-established long-term stability and sensitivity to subtle brain atrophy [27,28,29]. Recent studies suggest that synthetic 3D T1-weighted images derived from quantitative MRI can produce volumetric measures comparable to MPRAGE in cross-sectional analyses and short-term repeatability settings [6,30,31]. However, evidence supporting the reliability of synthetic MRI for multi-year longitudinal morphometry, including cross-time comparability and sensitivity to neurodegenerative atrophy rates, remains limited. Small systematic differences in tissue contrast or bias-field characteristics, while negligible cross-sectionally, may affect longitudinal atrophy estimates.

Therefore, future work should systematically evaluate synthetic MRI in multi-site, multi-vendor longitudinal settings, assess variability of derived quantitative biomarkers over long intervals, and determine harmonization strategies that ensure cross-time comparability comparable to established structural protocols. Such validation will be essential before synthetic imaging can be confidently adopted as a surrogate or replacement for conventional standardized sequences in long-term neuroimaging studies.

## 5. Limitations

This study has several limitations that should be considered when interpreting the results.

First, the cohort (*n* = 31) and the number of readers (*n* = 3) were limited, reducing statistical power and generalizability. This is particularly relevant for interrater variability analyses, where agreement was *poor* to *moderate* for several key parameters, especially for 3D SI. With few readers, variability estimates are less stable and may overemphasize individual rating tendencies, and with a modest sample size, subgroup effects and covariate influences may be missed. Accordingly, the reported agreement metrics should be interpreted as exploratory rather than definitive, and larger, multi-reader, multi-center studies are required to robustly quantify interrater reliability and its determinants for 3D SI.

Second, imaging protocols did not include optimized or pathology-specific sequences such as double inversion recovery (DIR), limiting the conclusions on the utility of 3D SI in specific diseases like MS.

Third, 3D SI was performed after cMRI, potentially benefiting from reduced patient motion as subjects became more relaxed.

Fourth, all scans were acquired on MRI systems from a single vendor using the same reconstruction software. This limits generalizability and raises concerns regarding reproducibility across vendors, scanner generations, and software versions. In particular, 3D SI depends on vendor-specific acquisition implementations and reconstruction pipelines (including denoising, intensity scaling, and synthetic contrast generation), which may lead to systematic differences in SNR, contrast behavior, and artifact patterns when transferred to other platforms. Therefore, the imaging characteristics and reader-dependent findings reported here may not directly translate to other vendors or to future software updates. Multi-vendor studies with harmonized protocols and explicit reporting of software versions are required to assess cross-platform robustness and to define clinically acceptable performance envelopes.

Fifth, contrast administration was inconsistent across patients and unbalanced between modalities. Because enhancement of extra-axial structures was apparent to readers, this may have introduced expectation bias and reduced comparability of subjective ratings. In addition, post-contrast FLAIR can modify the visual appearance of meningeal, vascular, and sulcal signals, which directly affects the parameters central to this study (CSF suppression, cortical surface appearance, and lesion suspicion near CSF interfaces). Given the small cohort, we could not robustly control for contrast timing effects, and residual confounding cannot be excluded.

Sixth, this study did not assess diagnostic accuracy or correlate imaging findings with clinical outcomes. Therefore, while image quality and reader confidence were evaluated, the clinical impact of 3D SI remains to be determined.

Seventh, we did not formally evaluate the effect of structured reader training or quantify learning curves over time. Given the reader-dependent ratings observed for 3D SI, the absence of longitudinal training data limits conclusions about how quickly radiologists can achieve stable interpretive performance and what minimum exposure is needed before routine clinical implementation.

Eighth and last, this study focused on subjective image quality and reader confidence rather than diagnostic accuracy. Without correlation to clinical outcomes, pathology, or an external reference standard, it remains unclear how observed differences in SNR and contrast translate into real-world diagnostic performance, including sensitivity, specificity, and error patterns for specific disease entities. Therefore, while perceived readability and contrast behavior were characterized, the clinical impact of 3D SI requires prospective diagnostic-accuracy studies with predefined tasks, reference standards, and outcome correlation.

## 6. Conclusions

In our study, cMRI outperformed 3D SI in both subjective quality and contrast perception. However, 3D SI showed promise, particularly in readability and clinically relevant tissue parameters. It can be judged as having acceptable-to-excellent quality. However, due to its reduced consistency in structural contrast, FLAIR imaging may currently limit diagnostic applicability.

Looking ahead, image interpretation may move increasingly toward non-visual, AI-driven analysis. In this context, 3D SI may represent an intermediate step toward a more automated and fully digital diagnostic workflow. However, until such tools are fully validated and integrated into clinical practice, understanding the current strengths and limitations of synthetic imaging remains crucial for safe and effective patient care.

## Figures and Tables

**Table 1:** Summary of the imaging characteristics and sequences evaluated for both conventional MRI and 3D synthetic imaging. For each parameter, a short description and the corresponding evaluation scale are provided. FLAIR, fluid-attenuated inversion recovery. The “x” is equal to a checkmark. It indicates the presence (in the row and line).

Characteristic	FLAIR	T2w	T1w	Scale	Mind-Map Quartiles
Signal-to-Noise	x	x	x	Low–high, 0 to100.	poor < sufficient < good < excellent
Overall Contrast	x	x	x	Poor–excellent, 0 to 100.	poor < sufficient < good < excellent
Correctly Suppressed/Homogeneous Cerebral Fluid in Sulci and Cisterns	x	x	x	Insufficient = 0 to fully suppressed = 100.	poor < sufficient < good < excellent
Signal in the Basilar Artery			x	Signal in prepontine basilar artery 0 to 100: when equal to cerebral fluid = 0, when equal to fat max = 100.	black < dark gray < light gray < white
Signal in the Basilar Artery		x		Signal in prepontine basilar artery 0 to 100: when equal to cerebral fluid = 100, when equal to air = 0.	white < light gray < dark gray < black
Signal in Sagittal Sinus		x	x	0 to 100: signal in sagittal and confluence venous sinus: signal T1w when equal to fat = 0, when equal to cerebral fluid = 100; signal T2w equal to cerebral fluid = 0, when equal to air = 100.	white < light gray < dark gray < black
Cerebral Fluid Homogeneity in Lateral Ventricles	x	x	x	0 to 100, diffuse heterogenic signal = 0, homogeneous signal = 100	mostly heterogenic, more than half of the volume heterogenic, mostly homogeneous, homogeneous
Spacial Accuracy in Respect to Motion Distortion			x	0 to 100, insufficient for clinical diagnosis = 0, no motion detectable = 100	poor < sufficient < good < excellent
Cortex Versus White Matter Contrast	x	x	x	0 to 100, no contrast detectable = 0, excellent contrast detectable = 100	poor < sufficient < good < excellent
Contrast of Lentiform Nucleus Against Surrounding Tissue	x	x	x	0 to 100, no contrast detectable = 0, excellent contrast detectable = 100	poor < sufficient < good < excellent
Contrast of Fluid in Sulci Against Cortex Tissue	x	x	x	0 to 100, cerebral fluid signal completely effaced = 0, excellent contrast sharpness of cerebral fluid against cortex in all sulci visible = 100.	poor < sufficient < good < excellent
Contrast of Lesions in White Matter Against Surrounding Tissue	x			0 to 100, if present: hyperintense white matter lesion contrast against white matter not distinguishable from noise = 0, excellent = 100.	poor < sufficient < good < excellent
Clarity of Remnant Cysts and Perivascular Spaces Versus White Matter Lesions		x		0 to 100, if present: confidence of recognizing remnant cysts and/or choroidal fissure cysts, when not certain = 0, maximally certain = 100.	poor < sufficient < good < excellent
Non-Concordant Lesions on FLAIR 3D Between SI and Conventional	x			yes, no	
Global Cortical Atrophy (GCA) Score			x	0–3	

**Table 2:** Multiple linear regression with signal-to-noise ratio (on 0–100 scale; labeled “Signal-to-Noise Ratio”) as outcome and T1w imaging methods as variable of interest, using conventional MRI as the reference.

Signal-to-Noise, T1w		Minimal Linear Regression Estimates (95% Confidence Interval)	Adjusted Linear Regression Estimates (95% Confidence Interval)
Method	Conventional MRI	Reference	Reference
	Synthetic MRI	−8.76 (−11.26 to −6.27, *p* < 0.001)	−8.76 (−11.26 to −6.27, *p* < 0.001)
Readers	R1	Reference	Reference
	R2	14.63 (11.57 to 17.69, *p* < 0.001)	14.63 (11.57 to 17.69, *p* < 0.001)
	R3	11.40 (8.35 to 14.46, *p* < 0.001)	11.40 (8.35 to 14.46, *p* < 0.001)
Scan Magnetic Field Strength	1.5 Tesla	-	Reference
	3.0 Tesla	-	6.73 (0.89 to 12.58, *p* = 0.012)
Thickness of Image Slices	1 mm	-	Reference
	1.2 mm	-	−0.04 (−14.74 to 14.66, *p* = 0.498)
	1.3 mm	-	2.55 (−5.10 to 10.21, *p* = 0.257)
	1.5 mm	-	−6.80 (−17.03 to 3.43, *p* = 0.096)
	1.7 mm	-	2.71 (−5.61 to 11.02, *p* = 0.262)

## Data Availability

The datasets and code generated and/or analyzed during the current study are not publicly available because the authors’ institution does not currently provide a data repository. Access to the raw data and analysis code may be granted by the corresponding author upon reasonable request.

## Supplementary Materials

The following supporting information can be downloaded at https://www.mdpi.com/article/10.3390/tomography12020013/s1. Table S1a: Summary of acquisition parameters of 3D SI for different voxel sizes. Table S1b: Summary of acquisition parameters of cMRI for different sequences. Table S2: Window settings for 3D SI and cMRI weightings.

## Abbreviations

The following abbreviations are used in this manuscript:

3D SI	three-dimensional synthetic magnetic resonance imaging
2D	two-dimensional
CSF	cerebral spinal fluid
CI	confidence intervals
cMRI	conventional magnetic resonance imaging
FLAIR	fluid-attenuated inversion recovery
fs	fat-saturated
GCA	global cortical atrophy
Hz	Hertz
ICC2	intraclass correlation
IQR	interquartile range
kg	kilogram
mg	milligram
min	minutes
mm	millimeter
MP-RAGE	magnetization-prepared rapid gradient-echo
ms	milliseconds
QALAS	Quantification using an interleaved Look–Locker acquisition sequence with T2 preparation
sec	seconds
SI	synthetic imaging
SNR	signal-to-noise ratio
T	Tesla
T1w	T1-weighted
T2w	T2-weighted
