# Supplementary material for: Clinical Image Quality and Reader Variability in 3D Synthetic Brain MRI Compared with Conventional MRI
Source: Tomography. 2026 Jan 23;12(2):13. doi: 10.3390/tomography12020013 (PMC12945194; doi:10.3390/tomography12020013)
Supplement: Supplementary file 1 [file tomography-12-00013-s001.zip › tomography-4078623-supplementary.pdf]

Supplementary Material

Table S1a Synthetic MR acquisition details

**Scan Parameters 3D SI**							
Field Strength/Tesla	3	3	3	3	3	1.5	1.5
3D SI Voxel size/mm	1.7	1.5	1.33	1.2	1.02	1.7	1.5
3D Acquisition Plane	sagittal	sagittal	sagittal	sagittal	sagittal	sagittal	sagittal
Field of View (mm)	256*256	240*240	240*240	249*249	245*249	272*272	240*240
Repetition Time (ms)	5.72	5.76	5.8	5.9	5.98	5.76	5.76
Echo Time (ms)	2..24	2.22	2.24	2.25	2.23	2.25	2.25
Inversion Time (ms)	110	110	110	110	110	110	110
Matrix in mm	144x144	160*160	192*192	208*208	240x240	160*160	160*160
Echo-Train Length	129	128	126	125	123	128	128
Flip Angle	4	4	4	4	4	4	4
Averages	1	1	1	1	1	1	1
Bandwidth (Hz/Pixel)	299	309	322	329	359	310	310
Acquisition Time (min,sec)	3,37	4,45	6,29	7,32	11,03	3,37	4,45

3D = three-dimensional, min = minutes, sec = seconds, ms = milliseconds, mm = millimeter, Hz = Hertz.

Table S1b. cMRI acquisition details

**Scan Parameters conventional MR sequences**						
Sequence	T2 3D	T2 3D	FLAIR 3D fs	FLAIR 3D fs	T1w MP-RAGE fs	Tw1 MP-RAGE fs
Field Strength/Tesla	1.5	3	1.5	3	1.5	3
Acquisition Plane	sagittal	sagittal	sagittal	sagittal	sagittal	sagittal
Field of View (mm)	250*250	250*250	240*240	250*250	250*250	250*250
Matrix (Frequency)	256	256	256	256	256	256
Matrix (Phase)	243	256	162	230	246	230
Repetition Time (ms)	3200	3200	5000	4900	2050	2060
Echo Time (ms)	381	412	334	388	2.7	3.2
Inversion Time (ms)	no	no	1600	1580	1100	1110
Bandwitdth (Hz/Pixel)	700	751	590	751	150	250
Section Thickness (mm)	1	1	1	0.9	1	0.4
Echo Train Length (ms)	236	260	218	255	1	1
Flip Angle in °	120	120	120	120	15	8
Number of Averages	2	1	1	2	1	1
Acquisition Time (min, sec)	3,27	3,51	4,57	4,54	4,43	4,28
Percent Sampling (%)	100	100	80	90	96	90

FLAIR= fluid-attenuated inversion recovery, MP-RAGE= magnetization-prepared rapid gradient-echo, 3D= three-dimensional, fs = fat saturated, min= minutes, sec = seconds, ms = milliseconds, mm = millimeter, Hz = Hertz.

Intern

Table S2. Window settings for 3D SI and cMRI weightings.

	Center	Width
3D SI FLAIR*	18.7	35.5
3D SI T1w*	37.2	63.3
3D SI T2w*	27	42
cMRI FLAIR**	1849	1064
cMRI T1w**	1837	1057
cMRI T2w**	1880	1081

FLAIR = fluid-attenuated inversion recovery; T1w = T1-weighted, T2w= T2-weighted;
*normalized units, **High Bit 11, Bit Allocated 16
